# Indigenous *Vibrio cholerae* strains from a non-endemic region are pathogenic

**DOI:** 10.1098/rsob.120181

**Published:** 2013-02

**Authors:** Atiqul Islam, Maurizio Labbate, Steven P. Djordjevic, Munirul Alam, Aaron Darling, Jacqueline Melvold, Andrew J. Holmes, Fatema T. Johura, Alejandro Cravioto, Ian G. Charles, H. W. Stokes

**Affiliations:** 1 The ithree institute, University of Technology, Broadway, Sydney, New South Wales 2007, Australia; 2 Enteric and Food Microbiology Laboratory, Laboratory Sciences Division, International Center for Diarrheal Disease Research, Dhaka, Bangladesh; 3 Genome Center, University of California Davis, 451 Health Sciences Drive, Davis, CA 95616, USA; 4 School of Molecular Bioscience, University of Sydney, Camperdown, New South Wales 2007, Australia; 5 International Vaccine Institute, San 4-8 Nakseongdae-dong, Gwanak-gu, Seoul 151919, Republic of Korea

**Keywords:** *Vibrio cholerae*, cholera, lateral genetic transfer, virulence, environmental

## Abstract

Of the 200+ serogroups of *Vibrio cholerae*, only O1 or O139 strains are reported to cause cholera, and mostly in endemic regions. Cholera outbreaks elsewhere are considered to be via importation of pathogenic strains. Using established animal models, we show that diverse *V. cholerae* strains indigenous to a non-endemic environment (Sydney, Australia), including non-O1/O139 serogroup strains, are able to both colonize the intestine and result in fluid accumulation despite lacking virulence factors believed to be important. Most strains lacked the type three secretion system considered a mediator of diarrhoea in non-O1/O13 *V. cholerae*. Multi-locus sequence typing (MLST) showed that the Sydney isolates did not form a single clade and were distinct from O1/O139 toxigenic strains. There was no correlation between genetic relatedness and the profile of virulence-associated factors. Current analyses of diseases mediated by *V. cholerae* focus on endemic regions, with only those strains that possess particular virulence factors considered pathogenic. Our data suggest that factors other than those previously well described are of potential importance in influencing disease outbreaks.

## 2. Introduction

*Vibrio cholerae* causes cholera. There are over 200 identifiable serogroups, which is just one manifestation of a highly variable genome whereby individuals can possess hundreds of subspecies-specific, or even clone-specific, genes [1]. This variability is driven by high rates of recombination and lateral gene transfer (LGT) [2]. With respect to the disease, cholera is primarily confined to endemic regions of the globe, mostly in south Asia, punctuated by outbreaks elsewhere as a consequence of natural or human-induced disruption, as evidenced most recently in Haiti and Zimbabwe [3,4]. In either case, the pathogenic strain is almost exclusively a member of only one of two serogroups, O1 or O139, with the former being the most common. Human disease does not only result from infection with O1/O139 isolates, and some non-O1/O139 *V. cholerae* isolates can cause outbreaks or sporadic cases of non-cholera gastroenteritis [5,6].

At any particular time in history, cholera pandemics appear to be mediated by specific strains that replace pre-existing pandemic strains. The sixth pandemic was mediated by strains of the classical biotype, but in the 1960s this was replaced by the El Tor biotype, responsible for the ongoing seventh pandemic [7]. Within the current pandemic, a new cholera serogroup, O139, arose in the early 1990s. The O139 variant evolved from a *V. cholerae* O1 El Tor progenitor strain via LGT with a non-O1 strain that, among other changes, included a serogroup switch from O1 to O139 [8]. Another notable recent evolutionary development has been the appearance of atypical or hybrid El Tor strains [7]. These belong to the *V. cholerae* O1 serogroup, but share genomic properties with El Tor and classical biotype strains, again a result of LGT. Irrespective of the differences between pandemic strains, all carry a suite of virulence genes, with cholera toxin (*ctx*) and the toxin co-regulated pilin (*tcp*) genes the most crucial to disease causation [9]. The presence of these genes is considered essential in defining the toxigenic cholera strains that give rise to the classic rice stool diarrhoea [9].

Outbreaks of cholera in non-endemic regions are generally believed to be caused by importation from endemic regions. Most recently, this has been argued for the Haiti outbreak [10,11]. Even where outbreaks may occur via strain importation the rapid spread of strains in a contemporary global setting means that many disparate locations may acquire single or near-clonal lines very quickly, makes identifying the original source of an outbreak strain difficult [12]. Recent additional genomic analysis, for example, has suggested that more than one strain was present in the early outbreak in Haiti, with a high proportion of cases potentially being caused by non-O1/O139 strains [13].

The above recent observations reinforce the fact that there is much that is not understood about the genetics and epidemiology of cholera. Within and beyond endemic regions, a plethora of *V. cholerae* strains exist in the natural environment, yet apparently only toxigenic serogroup strains belonging to O1 and O139 predominate in causing cholera [5]. Furthermore, disease outbreaks are very dependent on environmental and seasonal factors [14]. Thus, while non-O1/O139 strains are ubiquitous and isolated year-round from a variety of aquatic ecosystems, the pandemic strains are rarely, if ever, isolated from surface waters of endemic regions even during seasonal outbreaks, in part because toxigenic strains may be present in a non-culturable state [15]. Even where toxigenic *V. cholerae* are found in natural aquatic ecosystems these can expel the cholera toxin (CT) phage and become CT^−^ [16]. Overall, the development of large outbreaks is argued to be a result of the interplay of local environmental factors with the mixing of genes via LGT between *V. cholerae* strains in the environment, which gives rise to epidemic strains [5,17]. This logic is largely predicated on the assumption that the causative agent is one of a limited number of clones harbouring particular suites of virulence or virulence-associated factors.

Studies seeking to elucidate the evolution of pandemic *V. cholerae* often include the study of environmental isolates from endemic regions. Among other issues, it is clear that such isolates possess some but not a majority of the virulence factors (or pathogenicity islands that carry them) that are associated with epidemic strains. Of the ones that may be present, *toxR*, *hlyA* and to a lesser extent *ompU* are common [5,18]. This is true of both O1 and non-O1/O139 strains. Recently, other genes or gene combinations have been implicated in pathogenicity in non-O1/O139 serogroup strains. Most notable among these is the suite of genes comprising the type three secretion system (T3SS) [19], which is essential for virulence in some non-O1/O139 strains (six) and represents a diarrhoeagenic mechanism distinct from that characteristic of toxigenic O1 and O139 strains. However, this may not be the only mechanism for pathogenicity in non-O1/139 strains. Other important virulence factors include Cholix toxin (ChxA), an ADP-ribose transferase and a potent cytotoxin [20]. Finally, the haemagglutinin/protease (HA/P) is important in the spread of cells through the gastrointestinal tract [21].

Many environmental strains, including those of non-O1/O139 serogroups, can be closely related to pandemic strains based on fingerprint analyses. This reinforces the point that aquatic environments are likely to be habitats where pathogenic strains emerge from their non-pathogenic progenitor(s) or, by acquiring genetic material by LGT, create new pandemic strains [22,23]. However, the lack of most virulence factors, especially the *ctx* toxin genes and the colonization factor *tcpA*, is generally taken to imply that such environmental strains are non-virulent even where this may not have been specifically tested in animal models [24]. *Vibrio cholerae* is autochthonous to marine and estuarine environments generally, including in non-endemic areas [23]. Thus, understanding the evolution of epidemic cholera requires an understanding of the relationship between environmental strains in non-endemic regions compared with their more studied endemic counterparts. This is especially important since LGT is believed to readily occur between pandemic and environmental *V. cholerae*.

## 3. Material and methods

### 3.1. Collection of environmental samples and selection of *Vibrio cholerae* colonies

Water samples (50 ml) were collected from four locations in the greater Sydney (Australia) urban area in the period August 2009 to December 2010 (table 1). Samples were transported and processed as described previously [25].

**Table 1.** Collection site data. GR, Georges River at East Hills; SPC, Salt Pan Creek at Riverwood; RC, Redfern Creek at Ingleburn; MC, Muddy Creek at Rockdale.

site	location	sample date	number screened	*ompW* positive^a^
GR	33°57.9′ S, 150°58.9′ E	30 Aug 2009	152	0
		8 Sep 2009	105	0
		15 Sep 2009	172	5 (S10, S11, S12, S16, S18)
		23 Sep 2009	90	1 (S22)
		11 Oct 2009	41	1 (S23)
		13 Sep 2010	122	1 (S25)
		20 Oct 2010	70	1 (S29)
		21 Dec 2010	568	0
SPC	33°57.1′ S, 151°02.5′ E	30 Aug 2009	95	0
		8 Sep 2009	75	0
		15 Sep 2009	102	0
		23 Sep 2009	62	0
		13 Sep 2010	53	0
		20 Oct 2010	53	0
		21 Dec 2010	498	0
RC	34°00.2′ S, 150°52.0′ E	30 Aug 2009	47	0
		8 Sep 2009	38	0
		15 Sep 2009	60	0
		23 Sep 2009	0	0
		11 Oct 2009	3	0
MC	33°57.4′ S, 151°08.3′ E	30 Aug 2009	22	0
		8 Sep 2009	17	0
		15 Sep 2009	33	0
		23 Sep 2009	0	0
		11 Oct 2009	5	0

^a^Designations in brackets refer to individual samples as described in the text.

### 3.2. PCR amplification of *Vibrio cholerae* genes

Genomic DNA was extracted from overnight culture using genomic DNA extraction kit (Promega, catalogue no. A1125) according to the manufacturer's instructions. Isolated DNA was diluted 100-fold in ultrapure water, and 2 µl of diluted DNA was used in a final volume of 20 µl PCR reaction and was amplified in a PCR Mastercycler (Eppendorf GA 22331, Germany). Primers used for PCR and MLST analysis are shown in table 2. PCR screening conditions for *wbe*, *wbf*, *ctxA* were according the methods of Alam *et al.* [14] and *ompW* was according to the method of Nandi *et al*. [26]. PCR conditions for the T3SS genes were the same except that an annealing temperature of 59°C was used. The remaining virulence-associated genes were amplified individually according to the conditions listed in the references in table 2.

**Table 2.** PCR and MLST primers.

target	nucleotide sequence (5′-3′)	amplicon size (bp)	reference
ompWF ompWR	CACCAAGAAGGTGACTTTATTGTG GGTTTGTCGAATTAGCTTCACC	304	[26]
ctxA-F ctxA-R	CTCAGACGGGATTTGTTAGGCACG TCTATCTCTGTAGCCCCTATTACG	302	[14]
wbeO1F wbeO1R	GTTTCACTGAACAGATGGG GGTCATCTGTAAGTACAAC	192	[14]
wbfO139F wbfO139R	AGCCTCTTTATTACGGGTGG GTCAAACCCGATCGTAAAGG	449	[14]
hlyA-F hlyA-R	AGATCAACTACGATCAAGCC AGAGGTTGCTATGCTTTCTAC	1677	[27]
zot-F zot-B	TCGCTTAACGATGGCGCGTTTT AACCCCGTTTCACTTCTACCCA	947	[28]
ace-F ace-B	TAAGGATGTGCTTATGATGGACACCC CGTGATGAATAAAGATACTCATAGG	316	[28]
ompU-F ompU-B	ACGCTGACGGAATCAACCAAAG GCGGAAGTTTGGCTTGAAGTAG	869	[28]
tcpA-F tcpA-B/C	CACGATAAGAAAACCGGTCAAGAG TTACCAAATGCAACGCCGAATG	620	[28]
tcpA-F tcpA-B/E	CACGATAAGAAAACCGGTCAAGAG CGAAAGCACCTTCTTTCACACGTTG	453	[28]
toxR-F toxR-B	CCTTCGATCCCCTAAGCAATAC AGGGTTAGCAACGATGCGTAAG	779	[28]
stn/sto-F stn/sto-R	TCGCATTTAGCCAAACAGTAGAAA GCTGGATTGCAACATATTTCGC	172	[29]
PilE-F PilE-R	CATACCTTTTGAGCATCGAC GTGGCAAGAAGGACTCG	3087	[27]
rstC-F rstC-R	AACAGCTACGGGCTTATTC TGAGTTGCGGATTTAGGC	238	[27]
acfB1 acfB2	GATGAAAGAACAGGAGAGA CAGCAACCACAGCAAAACC	1180	[27]
rstA1 rstA2	ACTCGATACAAACGCTTCTC AGAATCTGGAAGGTTGAGTG	1009	[27]
rtxA1 rtxA2	GCGATTCTCAAAGAGATGC CACTCATTCCGATAACCAC	1366	[27]
msh1 msh2	AAAAGTCGACAGCGAAAGCGAATAGTGG AAAAGGATCCATTGCACCAGCAACTGCACC	380	[30]
tcpI-F tcpI-R	TAGCCTTAGTTCTCAGCAGGCA GGCAATAGTGTCGAGCTCGTTA	862	[29]
rstR-2F (ET) rstA-3R	GCACCATGATTTAAGATGCTC TCGAGTTGTAATTCATCAAGAGTG	501	[31]
rstR-1F (C) rstA-3R	CTTCTCATCAGCAAAGCCTCCATC TCGAGTTGTAATTCATCAAGAGTG	447	[31]
mdh-F mdh-R	GATCTGAGYCATATCCCWAC GCTTCWACMACYTCRGTACCCG	452	[32]
adk-F adk-R	GTATTCCACAAATYTCTACTGG GCTTCTTTACCGTAGTA	463	[32]
gyrB-F gyrB-R	CGTTTYTGGCCRAGTG TCMCCYTCCACWATGTA	713	[32]
recA-F recA-R	TGGACGAGAATAAACAGAAGGC CCGTTATAGCTGTACCAAGCGCCC	618	[32]
vcsC2-F vcsC2-R	GCCTAAAAACATCTCACCAG TCTTCAAGAAGTCGGTTGTT	671	this study
vspD-F vspD-R	AAATGACCTTTGGCGTACTA ATTGACTTCCATTTGTTTGG	739	this study
vcsN2-F vcsN2-R	GACGTTTTTGTTTTCCTTTG TTTCAAGATCATTGCCTTTT	931	this study
vcsV2-F vcsV2-R	GCGATGAAATTTGTTAAAGG CCTTCAAGTCACAATCCAAT	601	this study
chxA-F chxA-R	TGGTGAAGATTCTCCTGCAA CTTGGAGAAATGGATGCGCTG	421	[20]
HA/protease-F HA/protease-R	ACGTTAGTGCCCATGAGGTC ACGGCAAACACTTCAAAACC	350	[21]

### 3.3. Phylogenetic inference and recombination analysis

**Figure 1.**
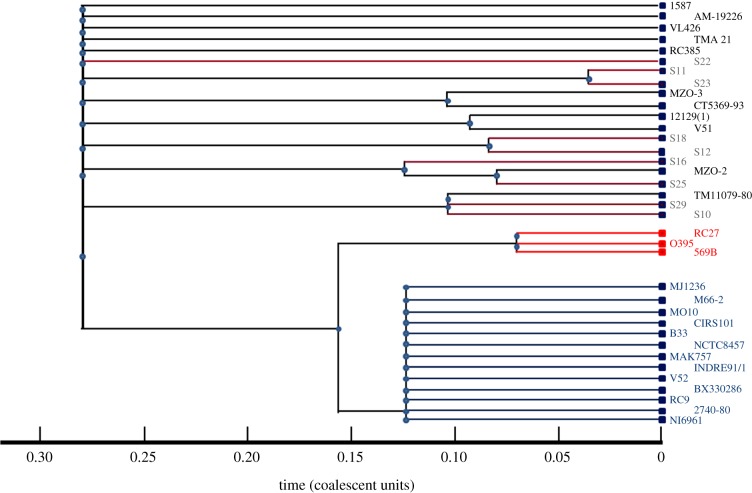
Phylogenetic tree of Sydney *Vibrio cholerae* strains. Strains in green: *V. cholerae* O1 El Tor or El Tor hybrid clinical isolates; red: *V. cholerae* O1 classical clinical isolates, grey: Sydney environmental isolates. The remaining strains comprise clinical and environmental isolates of various serogroups. DNA sequences of non-Sydney isolates were sourced from database entries with the following accession nos.: 1587: NZ_AAUR00000000.412966; AM-19226: NZ_AATY00000000.404974; VL426: NZ_ACHV00000000.593585; TMA21: NZ_ACHY00000000.593590; RC385: NZ_AAKH00000000.345074; MZO-3: NZ_AAUU00000000.41883; CT5369-93: NZ_ADAL00000000.675809; 12129(1): NZ_ACFQ00000000.592313; V51: NZ_AAKI00000000.345075; MZO-2: NZ_AAWF00000000.417398; TM11079-80: NZ_ACHW00000000.593586; O395: NC_009457; RC27: NZ_ADAI00000000.675807; MJ1236: NC_012668; N16961: NC_002505; M66-2: NC_012578; B33: NZ_ACHZ00000000.417400; CIRS101: NZ_ACVW00000000.661513; MO10: NZ_AAKF00000000.345072; NCTC8457: NZ_AAWD00000000.417399; MAK757: NZ_AAUS00000000.412967; INDRE91/1: NZ_ADAK00000000.675808; V52: NZ_AAKJ00000000.345076; BX330286: NZ_ACIA00000000.593587; RC9: NZ_ACHX00000000.593589; 2740-80: NZ_AAUT00000000.412614.

We extracted homologous sequences from the loci *adk*, *gyrB*, *mdh* and *recA* from finished and draft genomes available from NCBI as of October 2012. These genomes are listed in figure 1. The same genes were recovered from all nine Sydney strains and 569B by PCR using the *adk*, *gyrB*, *mdh* and *recA* primers indicated in table 2, and the products sequenced. Sequences for the Sydney isolates are deposited in GenBank under accession nos. KC492996–KC493004 (*adk*), KC493005–KC493013 (*gyrB*), KC493014–KC493022 (*mdh*) and KC493023–KC493031 (*recA*). A multiple alignment of sequences generated by us and those extracted from genomes was constructed using MUSCLE v. 3.8 software [33]. Draft genomes found to be lacking sequences for any of the four loci were excluded from the analysis. Some draft genomes contained only partial sequences; these were included with missing data coded as gap characters. Three (N16961, MJ1236 and O395) of the finished genomes were included twice in the dataset as a control. The four multiple alignments were combined and used as input for Bayesian inference of genealogy and recombination events using ClonalFrame v. 1.2 software [34].

Given one or more multiple sequence alignments, ClonalFrame uses Markov chain Monte Carlo to co-estimate a phylogeny representing vertical inheritance, along with model parameters, including *theta* (the population mutation rate), *rho* (the population recombination rate), *delta* (the average length of recombined segments) and *nu* (the number of nucleotide substitutions introduced by each recombination event). We ran ClonalFrame three times with different random starting points and different pseudorandom number generator seeds. Each chain was run for 500 000 iterations with samples recorded every 100 iterations and the first 10 per cent of each chain discarded as burn-in. Convergence was assessed by visualizing the log-likelihood trace of each chain along with calculation of the potential scale reduction factor (PSRF) for the continuously valued model parameters [35]. In all cases, the PSRF was 1.0 ± 0.01, suggesting strong convergence among the three chains. Finally, each of the three independent chains arrived at the same consensus tree topology with highly similar branch length estimates.

### 3.4. Bacterial strain growth and secretome harvest

Duplicate *V. cholerae* strains were grown to stationary phase (16–18 h) in 20 ml of LB5 and cells removed by centrifugation (5000*g* for 20 min). The supernatant was filter sterilized and then subject to ultracentrifugation (170 000*g* for 3 h at 4°C) for removal of membrane vesicles. The proteins in the resulting supernatant were concentrated to a final volume of 5 ml using Vivaspin 5 kDa spin columns (GE Healthcare Life Sciences), reduced and alkylated with 5 mM tributylphosphine and 20 mM acrylamide for 90 min at room temperature [36], and then precipitated with acetone. The resulting protein pellet was resuspended in 2 M urea and 100 mM ammonium hydrogen carbonate. A total of 60 μg of protein was separated by SDS-PAGE and stained initially with Flamingo Fluorescent Gel Stain (Bio-Rad) followed by colloidal Coomassie Blue G-250. Unstained Precision Plus Protein Standards (Bio-Rad) were run on all polyacrylamide gels. Gel lanes containing the resolved secretome proteins were sliced into eight equal-sized pieces and then destained. Proteins were identified by in-gel trypsin digestion followed by LC-MS/MS as described previously [37].

### 3.5. Rabbit ileal loop assay

The rabbit ileal loop assay was carried out based on the method described previously [5] with some modifications. The experiments with rabbits were performed in two different phases in complete accordance with icddr,b ethical guidelines using male New Zealand white rabbits (2.5–3.0 kg). Seven loops of 7–9 cm were created in each animal by ligation while preserving intestinal blood supply. In the first phase of the experiment, the first six loops were inoculated with 1 ml of bacterial suspension of different test strains in PBS adjusted to 10^5^ CFU ml^−1^ [38]. The last loop was used as negative control and was inoculated with 1 ml of PBS alone. In the second phase of the experiment, the second, third, fifth and sixth loops were inoculated with 1 ml of bacterial suspension of the same strain in PBS adjusted to 10^5^ CFU ml^−1^ and the remaining three loops were inoculated with PBS alone. The volume of fluid accumulated in each loop after 18 h was measured and the fluid accumulation (FA) index was expressed as volume (ml)/length (cm). Ilial fluid was collected aseptically and selective plating was done to confirm the viable growth of target strains. Results were recorded as positive for FA in loops with an FA index greater than or equal to 0.5 and negative control loops accumulated no fluid [39].

### 3.6. Mouse colonization competition assays

Colonization abilities of the Sydney cohort of *V. cholerae* strains were tested in an infant mouse model (in complete accordance with icddr,b ethical guidelines) as described previously [5]. The competition assay was carried out using S18 as the competing reference strain as this was found to be naturally streptomycin-resistant. All other strains are streptomycin-sensitive. Cultures of each of the test strain and S18 were mixed in 1 : 1 ratio and 100 µl from approximately 10^6^ CFU ml^−1^ of the bacterial mix was orogastricaly fed to a group of five 5-day-old Swiss albino mice. After 18 h, the mice were euthanized. The intestinal contents of these mice were collected aseptically in a falcon tube containing 2 ml of PBS (pH7.4) and were homogenized. Serial dilutions of the homogenates were plated on LB agar containing streptomycin (100 µg ml^−1^) and on plates devoid of the antibiotic to determine the ratio of the test and streptomycin resistant competing S18 strain. Competitive index (CI) was determined by the formula CI = *T*_C_ – *T*_R_/*T*_R_, where *T*_C_ is the total number of cells on plates without streptomycin and *T*_R_ is the total number of cells of the streptomycin-resistant reference strain, S18, as determined on streptomycin-supplemented plates. Thus, CIs less than or greater than one imply a test strain that is, respectively, less or more competitive than the reference strain. Colonies were confirmed as *V. cholerae* by *ompW* PCR [26] of 20 randomly selected colonies from the plates lacking streptomycin across all samples.

## 4. Results

### 4.1. Strain isolation

Australia is a cholera non-endemic region. Cases are rare and result from infections having occured overseas, or from ingestion of contaminated imported food stuffs [40]. To investigate the types of indigenous strains in Australia, *V. cholerae* was isolated from surface water samples in the greater Sydney urban area in the period August 2009 to October 2010 (table 1). In total, 2483 presumptive *V. cholerae* isolates were obtained from TCBS agar plates and were tested by PCR for the *V. cholerae* species-specific gene *ompW* [26], with nine strains being positive (tables 1 and 3). All nine confirmed *V. cholerae* isolates came from only one of the four sites—Georges River (table 1). These nine *V. cholerae* strains were further screened for belonging to serogroup O1 or O139 by both serological assays and PCR. Using O1- and O139-specific monoclonal antibodies, none of the nine strains were positive for O139, but one (S29) was positive for serogroup O1. Thus, S29 was confirmed to be a *V. cholerae* O1 strain while the other strains were in non-O1/O139 serogroups. PCR analysis results were consistent with the serological results (table 3).

**Table 3.** The presence of pandemic and virulence-associated genes in *V. cholerae* strains isolated from environmental water samples.

strain	*ompW*	serogroup specific		virulence-associated genes					type III secretion system genes				*HA/P*	cholix toxin
		*wbeO1*	*wbfO139*	*ompU*	*toxR*	*hlyA*	*rtxA*	*mshA*	*vcsC2*	*vspD*	*vcsN2*	*vcsV2*		*chxA*
S-10	+	−	−	−	+	+	+	+	+	+	+	+	+	−
S-11	+	−	−	+	+	+	+	+	−	−	−	−	+	+
S-12	+	−	−	−	+	+	+	−	−	−	−	−	+	+
S-16	+	−	−	+	+	+	+	+	−	−	−	−	+	+
S-18	+	−	−	+	+	+	+	−	−	−	−	−	+	+
S-22	+	−	−	+	+	+	+	+	−	−	−	−	+	+
S-23	+	−	−	+	+	+	+	+	−	−	−	−	+	+
S-25	+	−	−	−	+	+	+	−	+	−	+	+	+	+
S-29	+	+	−	+	+	+	+	+	+	−	+	+	+	+
EDC002	+	−	−	+	+	+	+	−	−	−	−	−	+	+
N16961	+	+	−	+	+	+	+	+	−	−	−	−	−	−
569B	+	+	−	+	+	+	+	+	−	−	−	−	−	−

Plus and minus symbols (+/−) indicate the presence/absence of a gene-specific PCR product of the predicted length or protein. Other virulence-associated genes tested for by PCR but which were absent in all Sydney isolates were: *ctxA, ace, zot, rstR, rstA, rstC, tcpA, tcpI, acfB* and *stn*.

### 4.2. Pathogenicity assays

All nine isolates were tested for pathogenicity with respect to their ability to cause FA in a rabbit ileal loop model (§3). Remarkably, all strains except S10 had an FA index above 0.5 (table 4**)**, the accepted cut-off for FA in pathogenic strains [39]. S29, the single O1 strain, had an FA index of 1.8, which was higher than the pandemic *V. cholerae* El Tor reference strains N16961 (FA 1.5). The non-O1/O139 strains S18 (1.6) and S22 (1.5) also had a high FA index consistent with values for pandemic strains.

**Table 4.** FA and infection. n.a., not applicable; n.d., not determined.

strain	serogroup or biotype	rabbit ileal loop		infant mouse
		FA mean^a^	FA range	CI^a,b^
N16961	O1/El Tor	1.5 (2)	1.4–1.6	1.2 (3)
S10	non-O1/O139	0.4 (2)	0.4–0.4	0.4 (4)
S11	non-O1/O139	1.1 (2)	1.1–1.1	0.8 (4)
S12	non-O1/O139	0.7 (7)	0.6–1.0	0.6 (3)
S16	non-O1/O139	0.9 (2)	0.9–0.9	1.1 (3)
S18	non-O1/O139	1.6 (6)	1.3–1.7	n.d.
S22	non-O1/O139	1.5 (6)	1.3–1.6	1.1 (3)
S23	non-O1/O139	1.1 (2)	0.9–1.4	0.6 (4)
S25	non-O1/O139	0.9 (2)	0.8–1.0	0.7 (4)
S29	O1	1.8 (2)	1.7–1.9	0.9 (3)
EDC002	non-O1/O139	0 (6)	n.a.	0.2 (5)
PBS	no cells	0 (12)	n.a.	n.a.

^a^Number of replicates in brackets. For the infant mouse model, this corresponds to the number of mice euthanized after 18 h.

^b^Competition index was determined using S18 as the reference strain (see §3).

In separate experiments, the strains were tested for pathogenicity with respect to their ability to colonize the gut via intra-gastric inoculation in the mouse model (see §3; table 4). This was done by a competition assay taking advantage of the fact that strain S18 is naturally streptomycin-resistant, whereas all the other strains were sensitive to this antibiotic. A panel of eight of the Sydney isolates was used in competition assays against S18, using the pathogenic strains N16961 as a positive control and the benign non-colonizing strain EDC002 as a negative control. The inoculum size used was approximately 2.0 × 10^5^ cells. Output population sizes were all in the range of 10^8^–10^9^, cells indicating that at least one of the strains was capable of growth in the gut after transit from the mouth. For the control strains, the CIs were broadly consistent with their known pathogenic potential. For example, the pandemic strain N16961 had a high CI (1.2), whereas the environmental non-pathogenic strain EDC002 had a low CI (0.2). The Sydney test strains had CIs consistent with the rabbit FA data. For example, S11, S22 and S29 had high FA scores and their respective CIs were close to one, this latter score for each implying an ability to efficiently colonize. By contrast, S12, S25 and especially S10 had lower values in both assays. The one anomaly was S16, which had a low (but still significant) FA score, but a high CI.

Using established criteria [39], eight of the nine Sydney strains scored as positive for FA potential as well as being able to proliferate on intestinal epithelial cells after passage through the acid environment of the murine stomach. Only one strain, S10, was non-pathogenic by these tests. Our data support the hypothesis that the non-endemic region of Australia harbours environmental isolates of *V. cholerae* that are as virulent in two animal models as O1 and O139 human pandemic strains.

### 4.3. Virulence gene profile

All nine strains were screened by PCR for the presence of 15 genes commonly associated with virulence from O1 serogroup strains. These genes were *ctxA, ace, zot, rstR, rstA, rstC, tcpA, tcpI, acfB, stn, ompU, toxR, hlyA, rtxA* and *mshA* (table 3). None of the strains were positive for *ctxA* encoding the major CT. They were all similarly negative for *zot*, the product of which can potentially increase the permeability of rabbit ileal mucosa by affecting the structure of the intracellular tight junction. However, they all did possess *hlyA,* a gene whose product is an exotoxin related to CT, and *rtxA*, a heat-stable enterotoxin, both of which can be found in non-O1 strains isolated from patients with cholera [41], or from environmental strains from endemic areas [5,24,42]. All strains possessed *toxR,* a 32-kDa transmembrane protein that acts as a master regulator of *ctxAB* gene [43]. Finally, six strains possessed *ompU,* the product of which is implicated in colonization and which can also be found in some environmental isolates from endemic regions [18]. Of the factors important for colonization, all strains lacked *tcpA*, a gene essential for colonization in O1 pathogenic strains, but several strains possessed *mshA*, encoding a type IV pilus that is also implicated in colonization. Overall, despite having characteristics in animal models consistent with toxigenic strains from endemic regions, the strains here lacked most of the genes described previously as being associated with such toxigenic strains.

### 4.4. The type three secretion system

The type three secretion systems (T3SSs) are widely distributed in Gram-negative bacteria, where they are frequently associated with virulence [44]. Recently, fatal diarrhoeal disease caused by non-O1/O139 strains of *V. cholerae* has been shown to be associated with a T3SS [6,45], a system absent in the common pandemic O1 strains. To test for the presence of a T3SS in the Sydney strains, PCR primer pairs were designed for four genes (*vcsC2*, *vcsN2*, *vcsV2* and *vspD*) within this system (table 3). Six of the nine Sydney strains were negative by PCR for all four genes, implying the absence of a T3SS in these strains (table 3). One strain, S10, was positive by PCR for all four genes tested while another two, S25 and S29, were positive for three genes but negative for one. In the Sydney isolates, there was no obvious correlation between the presence of a T3SS and pathogenicity in the animal models. For example, S10 was inferred to have a complete T3SS based on the presence of the four tested genes, but displayed only low pathogenicity (table 4), while the most pathogenic strains possessed only a partial T3SS (S29) or none at all (S18).

### 4.5. Presence and expression of cholix toxin

Two other genes, cholix toxin (*chxA*) and HA/P, associated with virulence in non-pandemic strains, were also tested for by PCR. It was found that the former was present in eight strains and the latter in all nine (table 3). Since the only Sydney strain lacking the *chxA* gene, S10, was the least pathogenic strain in the animal model experiments, we assayed for the presence of the ChxA protein in the secretome (§3) of all nine Sydney strains as well as EDC002, N16961 and 569B. Our preliminary proteomic analyses showed that ChxA was undetectable in S10, N16961 and 569B, which all lacked the gene. Of the remaining nine strains, including EDC002, which possessed the *chxA* gene, only S11 (Mascot score 573, seven peptides matched) had detectable levels of protein in the secretome (data not shown) under the nutrient growth conditions assayed.

### 4.6. Strain similarity and phylogenetic analysis

Genealogical analysis using the ClonalFrame v. 1.2 software yielded a partially resolved genealogy, with strong support for early divergence of the Sydney isolates from the other isolates in this study. A consensus tree of the genealogy is shown in figure 1. The Sydney isolates cluster into a small number of clades; however, the genealogical relationship among these clades is not well resolved by our data. We find two clades of Sydney isolates for which a near neighbour exists in the draft genome database. One of these groups comprises S10, S29 and TM11079-80. This last strain is an environmental isolate recovered from sewage in Brazil in 1980. Interestingly, it is described as a non-toxigenic El Tor isolate derived from a non-O1/O139 strain by lateral gene transfer (see database entry). Its potential to cause disease in humans and its actual pathogenicity in animals is unknown. A second clade comprises S16, S25 and MZO-2. MZO-2 was recovered from a patient with diarrhoea in Bangladesh in 2001. S16 and S25 are relatively diverse in their virulence gene profile despite being more closely related to each other than to other Sydney strains. For example, the former has *ompU* and *mshA* genes but no T3SS, whereas the latter lacks both *ompU* and *mshA* but does possess at least a partial T3SS.

Our MLST analysis implies that recombination introduces changes more frequently than *de novo* mutation in *Vibrio*. In the case of the four genes analysed here, the estimated ratio of recombination to mutation, r/m was 3.28 and the estimate for the average length of imported segments (*delta*) was 193.2 nucleotides. Relative to other Genera that have been studied, this estimated r/m for *V. cholerae* is neither high nor low. Many other commensal organisms, including members of *Haemophilus* and *Campylobacter*, have similar estimates for r/m [46]. Interestingly, other free-living species in the genus *Vibrio* have much higher estimated r/m, with *V. parahaemolyticus* at 39.8 and *V. vulnificus* at 26.7 [46]. This suggests a fundamental difference may exist in the recombination biology of *V. cholerae* relative to other *Vibrio* if the values found here apply more broadly across core genes in *V. cholerae*.

All nine were tested by ultra-fine-scale integron/gene cassette profiling. This method is highly sensitive and relies on the high rates of genome evolution of mobile cassette arrays to distinguish strains that may be indistinguishable by other typing methods [2]. Cassette profiling revealed fingerprints that were all so different from each other that the arrays can be considered to be randomly assorting (data not shown). This was true even for the pairs S10/S29, S11/S23 and S12/S18 when compared (figure 1). This finding further supports the idea that high rates of LGT have driven evolution in the Sydney strains at least with respect to non-core genes. Also, a prominent characteristic of the *Vibrio* spp. is the very large arrays associated with integrons [47] to a degree not seen in other genera [48]. High rates of LGT overall across the *Vibrios* may contribute to the accumulation of large numbers of cassettes in members of this genus.

## 5. Discussion

The primary symptom of cholera, rice water stool diarrhoea, can have a number of causes, even in regions where the disease is endemic [49]. While it is true that most diarrhoea cases in epidemics are caused by toxigenic *V. cholerae*, it is nonetheless the case that, where surveys take place, a large proportion of cases do not have a defined aetiology [50]. In one survey, the proportion of disease that could not be correlated with O1/O139 *V. cholerae* was reported at 45 per cent [49]. Difficulties with diagnosis are exacerbated by the fact that *V. cholerae* is an extraordinarily diverse species, subject to high rates of LGT whereby one or a limited number of events can bring about large phenotypic changes, as is the case for the O1–O139 switch [8]. This has ramifications for diagnostic testing since some markers used for that purpose do not absolutely correlate with pathogenic strains. This suggests that *V. cholerae* harbours pathogenicity determinants (e.g. toxins, colonization factors, etc.) that differ from those otherwise assumed essential for mediating disease [45]. It also suggests that the environment may harbour diverse *V. cholerae* strains with a range of disease potential, and that the significance of these organisms and their detection escapes current clinical screening. Our data show that a phylogenetically diverse panel of *V. cholerae* indigenous to a non-endemic setting of Sydney (including O1 and non-O1/non-O139 serogroup strains) is equally pathogenic as reference O1/O139 strains in animal models used to assess pandemic cholera strains. Although the genes (or pathways) mediating disease can vary, there is normally a very strong link between human disease strains and positive testing in animals. We therefore believe that these models can be informative in guiding experimental strategies.

In general, the virulence factor profile of the nine Sydney strains was similar to analogous environmental strains from endemic regions. Some key virulence factors such as *ctxA* and *tcpA* were absent, a fact that would normally lead to the conclusion that they are non-virulent, even if recovered from a region with endemic cholera and irrespective of any genetic similarity to pathogenic strains [24]. However, by testing all nine Sydney strains in the two animal models we were able to establish that the strains exhibited pathogenic behaviour. Eight of the nine strains were positive in both assays. While some non-O1/non-O139 strains lacking both the major toxin cholera gene *ctxA* and major colonization factor *tcpA* are known to mediate diarrhoea, such strains appear only rarely and have only been seen in endemic regions [6]. Non-O1/non-O139 strains have also been recovered from the environment that have been shown to mediate disease in animal models, but these, again, have only been identified in endemic regions and most commonly after enrichment through an animal [5].

One recent study has reported the recovery of *V. cholerae* from natural aquatic environments in Iceland [21]. This study was notable in that Iceland is a non-endemic cholera region and has never reported a case of the disease. The recovered strains were concluded to be autochthonous. Many sites were virtually free from human interference, implying the strains had no opportunity to have cycled through humans. Despite this, these strains were found to have a broadly similar virulence-associated gene profile to that seen here for the Sydney isolates. While it is not known if the Icelandic strains are pathogenic in animal models, we would predict that this is likely to be the case based on our findings. The sites sampled here were all within the greater Sydney urban environment. While less pristine than the Icelandic environment and impacted by recreational activities, the Sydney sites are nonetheless relatively free of environmental pollutants. Consequently, the findings reported here support the Icelandic study in suggesting that pathogenicity factors are commonly present in diverse environmental strains and that these factors may have an adaptive role for the bacterium in the natural environment.

What is the cause of the virulence in animal models of these Sydney strains? The strains are not equivalent to typical toxigenic strains from endemic regions as they all lack the major virulence factors *ctx* and *tcpA*. In addition, there is no correlation between other virulence-associated genes found in toxigenic strains and pathogenicity in the strains here. It has been shown that the presence of a T3SS can be a mediator of diarrhoeal disease in non-O1/O139 strains [6]. Interestingly, only one of the Sydney strains, S10, has a panel of T3SS genes based on PCR analysis (table 3), and this strain is the only one of the nine that is non-pathogenic based on animal model testing. Two strains, S18 and S29, showed levels of FA greater than the known pandemic strain N16961. These two strains are in different clades and are quite different based on their genetic profiles (table 3), to the point where they are also of different serogroups (S29 is an O1 strain and S18 is of another group). Neither strain, though, as for all other strains except S10, had all four T3SS genes screened for by PCR.

Recently, a new toxin gene, designated *chxA*, has been found in some strains of *V. cholerae*. This gene encodes a protein, designated cholix toxin, and is an ADP-ribosyl transferase that is cytoxic to mammalian cells [20]. Eight of the strains were positive by PCR for this gene and these were the eight strains that were also the most pathogenic in the animal models. However, in proteomic experiments, the Chx protein was detected in whole cell lysates in only S11 (table 3) under the nutrient growth conditions tested. It remains possible that more strains containing the *chxA* gene may conditionally express it under conditions related to animal–host interactions. However, even if this is the case it is unlikely on its own to account for the inferred pathogenicity seen here in animal models. ChxA is cytotoxic *in vitro,* and therefore lethal to animal cells, but this pathology would account for neither the colonization ability nor the FA seen here. Also, EDC002, a known non-pathogenic strain, possesses the *chxA* gene. Assuming, therefore, that strains which possess the gene can express it conditionally, it is perhaps more likely that ChxA has a role in survival in the general environment unrelated to animal infection directly.

The ability of any of the Sydney strains to actually cause disease in man is unknown. However, at least eight of the nine possess the phenotypes in animal models that are associated with human disease. These are colonization ability and FA. These eight strains achieve this despite lacking the virulence factors linked to these phenotypes in toxigenic strains, namely CtxA and TcpA. Most of the strains also lack a T3SS, the only other known system capable of mediating diarrhoeal disease by *V. cholerae* in humans.

Although *V. cholerae* is widely distributed in a range of natural surface water ecosystems [23], understanding the evolution of *V. cholerae* and the ecology of cholera outbreaks is complicated by a number of factors. One of these is LGT. This is particularly evident in this study, where the non-endemic Sydney strains showed a high degree of virulence and substantial evolutionary diversity. Furthermore, there is little congruence between the presence and the absence of particular virulence-associated genes and genetic relatedness based on MLST analysis. This lack of congruence extended to the apparent relationship of Sydney strains with other geographically diverse strains. For example, S10 and S29 fell within a clade with TM11079-80, a Brazilian environmental isolate from 1980 that is an O1 El Tor strain. The complete genome of this Brazilian strain implies that it was derived from a non-O1 strain by LGT. We also speculate that, in an analogous manner, strain S29 may be derived from a non-O1 strain as a result of LGT of the relevant serogroup genes. Such serogroup conversions have been shown experimentally, and this mechanism may explain the appearance of toxigenic O139 strains [51,52]. A search of the TM11079-80 genome for the virulence-associated genes listed in table 3 revealed that it did not absolutely correlate with either S10 or S29. Using the primers listed in table 2, it was predicted bioinformatically that the Brazilian strain would have been positive by PCR for *toxR*, *hlyA*, *rtxA*, *mshA* and HA/P, but negative for the rest of the genes tested (table 3), including the lack of a T3SS. While TM11079-80 does possess a predicted *ompU* gene, the sequence is sufficiently diverged such that it is unlikely to generate a product with the primers used here. This highlights the fact that relatively closely related strains can become globally dispersed while at the same time sharing a local environment with other strains that are more distantly related phylogenetically. This rich clonal diversity within an ecosystem also provides an opportunity for LGT of potential virulence traits for members of the genus *Vibrio*, especially where such transfer appears to be common. This is highlighted in the Sydney cohort, where phylogeny (figure 1) is not a good predictor of the presence/absence of virulence-associated traits, nor even of serogroup assignment (table 3). Also, even within a non-endemic region such as Australia, toxigenic-like *V. cholerae* strains are recoverable from the environment, as is the case, for example, for the strain BX330286 from 1986, which clusters tightly with the known pandemic strains (figure 1). Thus, the potential for LGT between pathogenic and non-pathogenic strains is likely to be a global phenomenon.

Other important issues impacting on our understanding of the generation of pathogenic strains include: seasonal variability and environmental factors [17,25]; symbiotic interactions with animals [53]; and the fact that toxigenic O1 and O139 serogroup strains can enter into a viable but non-culturable state that limits their ability to be detected by bacterial growth [54]. These factors confound a correlation of genotype with pathogenic properties [1]. Predicting future outbreaks of disease will require close monitoring for a more detailed picture of which *V. cholerae* strains can actually produce the disease. In particular, non-endemic region strains need to be examined more extensively and consideration given to the role of virulence traits beyond those normally considered important in disease manifestation. These points are especially critical to the likelihood of previously non-endemic regions becoming endemic regions with changing climate and habitats, and in assigning the true origins of geographically dispersed pathogenic strains.

## 6. Acknowledgements

This work was supported by the National Health and Medical Research Council of Australia (grant no. 488502) and the ithree institute, UTS. A.I. and J.M. are recipients of UTS post-graduate scholarships, and M.L. is a recipient of a UTS post-doctoral fellowship. We are grateful for the technical assistance of Dr Matt Padula in preparing and validating samples for LC-MS/MS. This research was also partly supported by icddr,b, which in turn is supported by donor countries and agencies, including AusAID, Govt. of Peoples Republic of Bangladesh, CIDA, Sida and DFID, that provide unrestricted support to icddr,b for its operation and research.
